# The effects of vitamin A supplementation during late-stage pregnancy on *longissimus dorsi* muscle tissue development, birth traits, and growth performance in postnatal Korean native calves

**DOI:** 10.5713/ajas.19.0413

**Published:** 2019-12-24

**Authors:** Yong Ho Jo, Dong Qiao Peng, Won Seob Kim, Seong Jin Kim, Na Yeon Kim, Sung Hak Kim, Jalil Ghassemi Nejad, Jae Sung Lee, Hong Gu Lee

**Affiliations:** 1 Department of Animal Science and Technology, Konkuk University, Seoul 05029, Korea; 2 Team of an Educational Program for Specialists in Global Animal Science, Brain Korea 21 Plus, Konkuk University, Seoul 05029, Korea; 3 Asia Pacific Ruminant Institute, Icheon 467814, Korea; 4 Department of Animal Science, Chonnam National University, Gwangju 61186, Korea

**Keywords:** Adipocyte Hyperplasia, Birth Weight, Korean Native Calves, Myogenesis, Vitamin A

## Abstract

**Objective:**

This study investigated the effects of vitamin A (VA) supplementation during late-stage pregnancy on *longissimus dorsi* muscle tissue development, birth traits, and growth performance of postnatal Korean native calves.

**Methods:**

In the preliminary experiment, twenty-six pregnant cattle (initial body weight [BW] = 319 kg (standard deviation [SD] = 30.1; 1st parity) were randomly assigned to the control and treatment groups. The treatment group received VA supplementation at 24,000 IU/d from gestational day 225 until delivery. In the main experiment, twelve pregnant cattle (initial BW = 317 kg [SD = 31.3]; 1st parity) were treated with VA supplementation at 24,000 IU/d (gestational days 150 to 225) and at 78,000 IU/d (gestational day 225 until delivery). Serum VA levels were analyzed in pregnant cattle, and the growth performance, gene expression, and serum VA levels were analyzed in the offspring.

**Results:**

Serum VA levels in pregnant cattle decreased the late gestation in both experiments (p<0.001). In the main experiment, pregnant cattle at parturition and offspring at birth in the treatment group had higher serum VA levels than those in the control group (p<0.05). In the treatment groups, an increased birth weight was observed in the main experimental group (p = 0.022), and a tendency (p = 0.088) toward an increased birth weight was observed in the preliminary experimental group. However, no differences were observed in the feed intake, average daily gain, gain-to-feed ratio, or BW of 31-day-old calves. Gene expression was analyzed in *longissimus dorsi* muscles of 31-day-old calves. VA supplementation in pregnant cattle stimulated postnatal muscle development in offspring by elevating myogenic factor 5 (*MYF5*), *MYF6*, and myoblast determination levels (p<0.05). Moreover, preadipocyte-related marker genes such as extracellular signal-regulated kinase 2 and krüppel-like factor 2 were higher in the treatment group than in the control group (p<0.05).

**Conclusion:**

VA supplementation (78,000 IU/d) in late-stage pregnant cattle maintained serum VA levels. In addition, 78,000 IU/d VA supplementation increased the birth weight and expression of genes related to muscle and preadipocyte development in offspring. Overall, 78,000 IU/d VA supplementation in pregnant cattle is beneficial to newborn calves.

## INTRODUCTION

In many countries, the production of beef with a high level of intramuscular fat contributes to consumer satisfaction and results in substantial benefits to producers [1]. According to a previous study [2], the increase in marbling stems from both intramuscular fat hypertrophy (increase in adipocyte size) and hyperplasia (increase in adipocyte number). Although numerous studies have pinpointed how adipocyte size can be increased [3,4], the question of how adipocyte number can be increased remains poorly understood.

A previous review [5] hypothesized that adipogenic and myogenic cells derived from the same pool of multipotent mesenchymal stem cells play a crucial role in enhancing carcass traits in cattle. Additionally, this hypothesis was tentatively confirmed in a mouse model; the results showed that adipogenic determination and preadipocyte proliferation are highly activity during the fetal and early growth stages [6]. Regulation by environmental and nutritional aspects during the fetal and early growth stages can alter organismal resilience to environmental stressors and the developmental process in humans and laboratory animals [7]. Previous research has indicated the effect of vitamin A (VA) on adipocyte hyperplasia and muscle development in cell lines, and primary cells [4,8]. However, to the best of our knowledge, an *in vivo* evaluation of the effects of VA supplementation in cattle in late pregnancy on their offspring has not yet been reported. Therefore, we hypothesize that VA supplementation during late-stage pregnancy can improve *longissimus dorsi* muscle tissue development, birth traits, and growth performance in offspring.

## MATERIALS AND METHODS

### Animals and management

All experimental procedures were in accordance with the “Guidelines for the Care and Use of Experimental Animals” of Konkuk University (Approval no: KU17099). Originally, fifty Korean native (Hanwoo) heifers were included in this research; however, twelve heifers were unexpectedly excluded due to premature birth, abortion or missed. Ultimately, thirty-eight 20-month-old heifers were selected for this study. In the preliminary experiment, twenty-six pregnant Hanwoo cattle (initial body weight [BW] = 319 kg (standard deviation [SD] = 30.1; 1st parity) were randomly allocated to two groups (high-VA group, n = 8; control group, n = 18). The same number of pregnant cattle were included in each group. However, it was difficult to control the experimental period during pregnancy. All the cattle were impregnated by artificial insemination, and the date of pregnancy was estimated by a commercial kit. As a result, nine cows had an inadequate treatment period due to an incorrect prediction of the delivery date, one cow experienced abortion during the preliminary experiment.

We supplied diets with total mixed ration (TMR). Animals were placed in a large pen with a Kalan gate in which clean dry sawdust was spread onto the floor for bedding, and water was available *ad libitum*. All pregnant cattle in the control and treatment groups were fed 13 kg of TMR feedstuff (on an as-fed basis) once a day at 0900 h (Table 1). None of the pregnant cattle remained on a TMR diet during the experiment (data not shown). The TMR contained VA (1,662 IU per kg) on an as-fed basis. The total amount of VA feeding was 21,606 IU per day from the TMR diet. In the treatment group, an additional 24,000 IU VA (retinyl acetate, Hanyou Feed, Wuxi, China) was supplied by oral administration for each heifer from day 225 of pregnancy until delivery. To achieve accurate VA supplementation, all the experimental pregnant cattle were locked with the stanchion gate, and 50 g of powdered VA was fed to the cattle in the treatment group. The total amount of VA feeding was 45,606 IU per day in the treatment group (Table 1).

In the main experiment, twelve Hanwoo cattle (initial BW = 317 kg [SD = 31.3]; 1st parity) in advanced pregnancy were randomly selected and allocated into two groups (high VA group, n = 5; control group, n = 7). We supplied the cattle with diets containing the same TMR feedstuff as the cattle in the preliminary experiment. All cattle were housed in the same conditions as those in the preliminary experiment, except the amount of VA supplementation differed in the treatment group. For the treatment group, each heifer was orally administered an additional 24,000 IU VA from 150 to 225 days of gestation (period 1) and 78,000 IU/d from day 225 of pregnancy until parturition (period 2; Table 1).

All newborn calves born to experimental cattle were weigh ed at birth. The birth weights averaged 26.9 kg (SD = 4.51) in the preliminary experiment and 23.7 kg (SD = 3.48) in main experiment. The average birth weight in the two experiments was similar to Korean Feeding Standard for Hanwoo (KFS [9]); the claves were immediately separated from their dams and randomly housed in individual wooden pens [200 cm (L)×135 cm (B)×120 cm (H)] with a stainless steel gate for one month. We provided individual buckets for both water and feedstuff for the calves. Clean dry sawdust, which was changed weekly, was spread onto the floor inside the pen for bedding. For newborn calves, 3.6 L of commercial colostrum (225 g in 625 mL of 45°C water, Headstart, Cat. No. 15360020, Saskatoon Colostrum Company Ltd., Saskatoon, SK, Canada) was given within 12 h after birth, followed by feeding with a commercial milk replacer at an approximate volume of 20% of the birth weight (140 g in 1 L, 45°C water, Nukamel Yellow, The Dawn Ltd., Seoul, Korea) until one month of age (Table 2). Calves who presented clinical diarrhea were treated with Biodyl, Baytril, and ketoprofen injections; and simultaneously administered a nutritional supplement formula (Life-aid, Norbrook, Northamptonshire, UK) with Neodiaristin.

The BWs of the calves were measured every week using a CAS for a month. We investigated the growth performance of the offspring for 31 days, and then, we collected muscle samples from the offspring. Biopsy causes considerable damage and stress to the calves, and this prevents the calves from growing normally.

### Preparation of blood samples

Blood samples were collected from the jugular vein of each pregnant heifer and calf by using 18-gauge needles at 0900 h. In the preliminary experiment, blood samples were collected via the jugular vein from each pregnant heifer at day 150 of pregnancy and 7 days after delivery. In the main experiment, blood samples were collected in the same way at days 150 and 225 of pregnancy and 7 days after delivery. Blood samples (15 mL) were obtained from each calf via the jugular vein at postnatal day 7 in the preliminary experiment and at postnatal days 7 and 31 in the main experiment. Each calf was born at a different time under different circumstances. Thus, a buffer period of one week was instituted for the calves and blood sampling was conducted at the same time in the morning. Serum tubes (wrapped in tin foil for VA analysis) and K2 ethylenediaminetetraacetic acid (EDTA) (K2E) 7.2 mg Plus Tubes (BD Vacutainer, Franklin Lakes, NJ, USA) were prepared, and the collected blood samples were stored on ice before being transferred to the laboratory. Then, the blood samples in the serum tubes were centrifuged at 2,740×g for 15 min at 4°C. Serum was transferred to a brown 1.5 mL tube and stored in a freezer (−80°C) before further analysis. The K2 EDTA blood sample was temporarily stored at 4°C for subsequent analysis.

### Serum vitamin A analysis

The serum VA analysis was performed with a previously described method [10]. In detail, the experiment was conducted in a dark room with red light to prevent oxidation. Retinyl acetate (>99%, Sigma-Aldrich R7882-1g, Yongin, Korea) was dissolved into 0.04% BHT-ethanol solution (2,6-di-tert-butyl-4-methylphenyl, Sigma-Aldrich, Cat. No. B1378, Yongin, Korea) as an internal standard (1 mg/mL, stock solution; 25 μg/mL, working solution). The ethanol, methanol, and hexane used in this study were of high performance liquid chromatography (HPLC) grade. After the serum samples were thawed at room temperature, 200 μL of serum and 20 μL of the internal standard working solution were added to a 2-mL microtube. Then, 200 μL of distilled water and 400 μL of 0.04% BHT-ethanol solution were added, and the solutions were thoroughly vortexed. After adding 800 μL of 0.04% BHT–hexane solution, the tube was vortexed vigorously again. The samples were then centrifuged at 3,500×g for 10 min at 4°C, after which 700 μL of the hexane layer was transferred to a new 1.5-mL brown microtube, followed by complete hexane evaporation under flowing N2 gas in a dry block bath (MG-2200, EYELA, Tokyo, Japan). The samples were dissolved in 95% methanol and mixed well by vortexing. Meanwhile, three controls (internal standards) were prepared in new 1.5-mL brown microtubes by mixing 20 μL of the internal standard working solution with 480 μL of 0.04% BHT-ethanol. Finally, the serum concentration of VA was analyzed by HPLC (Agilent 1100 series; Agilent Technologies, Waldbronn, Germany) in a brown LC vial (Infochroma AG, Goldau, Switzerland) after filtration with a syringe filter (PES, 0.33 mm, 0.22 μm, Millex, Darmstadt, Germany). A stainless steel Novapak C18 column (4-μm reversed-phase column, 3.9 mm ID×150 mm, Waters, Dublin, Ireland) was used in this system, with 95% methanol as the mobile phase at a flow rate of 1 mL/min. Serum VA (retinol) was detected by measuring the absorbance at 325 nm using a DAD-UV lamp. The column was set at 20°C. Peaks of retinol and retinyl acetate in the samples were obtained using an HPLC computer program. The standard curve was generated by diluting retinol (>99%, Sigma-Aldrich, Cat. No. R7632-25mg, Korea) to different concentrations (12.5, 6.25, 3.13, 1.56, 0.78, 0.39, 0.20, 0.10, and 0.05 μg/mL) with 1 μg/mL of the internal standard at each concentration. All serum VA samples were analyzed in duplicate.

### *Longissimus dorsi* muscle sampling and total RNA extraction

*Longissimus dorsi* muscle sampling and total RNA extraction were performed with minor modifications of previously described methods [10,11]. Tissue samples (approximately 2 g) were collected from the *longissimus dorsi* muscles of 31-day-old calves (n = 8) by biopsy. One calf in the treatment group had consistent diarrhea. Therefore, four calves were selected for the analysis of gene expression level. The biopsy causes considerable damage and stress to the calves, and this prevents the calves from growing normally. Therefore, we investigated the growth performance of the offspring for 31 days, and then, we collected muscle samples from the offspring. The biopsy procedure was in accordance with the “Guidelines for the Care and Use of Experimental Animals” of Konkuk University. Briefly, local anesthetic agents were administered at 6 equidistant points around the resection area, namely, the *longissimus dorsi* muscle at position 12 to 13 of the ribs. The veterinarian resected 5 cm of skin in the direction of the muscle and then dissected the *longissimus dorsi* muscle tissue using surgical equipment. The collected tissue was washed immediately with DEPC-treated distilled water (diethyl pyrocarbonate, Sigma-Aldrich, Cat. No. D5758-50ML, Korea), placed in liquid nitrogen until completely frozen, and placed on dry ice until transfer to the laboratory. Then, the tissue samples were ground into powder in liquid nitrogen. Total RNA was extracted by using TRIzol Reagent (Life Technologies, Cat. No. 15596-018, Seoul, Korea); 0.1 g of tissue sample was mixed with 1 mL of TRIzol. Next, the sample was homogenized by a homogenizer (IKA T10 basic, Seoul, Korea) and centrifuged at 4°C and 12,000×g for 10 min; then, the upper aqueous phase was transferred into a new 1.5 mL tube. Then, 200 μL of chloroform was added, and the mixture was incubated for 2 min at room temperature after vortexing, followed by centrifugation at 12,000×g for 15 min at 4°C. The upper transparent aqueous phase was transferred into a new 1.5 mL tube. Isopropanol (500 μL) was added, and the sample was incubated for 10 min at room temperature after vortexing, followed by centrifugation at 12,000×g for 10 min at 4°C to precipitate the RNA. Next, the supernatant was discarded, and the RNA was washed by adding 1 mL of 75% ethanol to the tube. After vortexing, the mixture was centrifuged at 4°C and 12,000×g for 10 min. To collect the RNA, the wash step was repeated with 1 mL of 100% ethanol following the same procedure. After washing, the extracted RNA was dried under a vacuum for approximately 15 min, redissolved in 30 μL of DEPC-treated distilled water, and then incubated at 60°C for 10 min. The RNA concentration was determined by spectrophotometric analysis (NanoDrop 1000, Thermo Scientific, Seoul, Korea). The RNA integrity was assessed using an RNA Nano 6000 Assay Kit for the Agilent Bioanalyzer 2100 system (Agilent Technologies, Richardson, TX, USA). Samples with an RNA integrity number (RIN) of 6 or higher were subjected to complementary DNA (cDNA) synthesis. The RNA used for real-time polymerase chain reaction (PCR) analysis had a RIN of 7.5 (SD = 0.4).

### Design of primers and probes

Bovine myoblast determination (*MyoD*), myogenic factor 5 (*MYF5*), myogenic factor 6 (*MYF6*), desmin, myogenie (*MyoG*), CCAAT/enhancer-binding protein alpha (*C/EBPα*), peroxisome proliferator-activated receptor gamma (*PPARγ*), lipoprotein lipase (*LPL*), stearoyl-CoA desaturase (*SCD*), extracellular signal-regulated kinase 1, 2 (*ERK1*, *ERK2*), Wnt family member 10B (*Wnt10B*), β-catenin, preadipocyte factor 1 (*Pref-1*), zinc finger protein 423 (*Zfp423*), *Sox9*, krüppel-like factor 2 (*KLF2*), 18S ribosomal RNA (*18S*), glyceraldehyde-3-phosphate dehydrogenase (*GAPDH*), and ribosomal protein lateral stalk subunit P0 (*RPLP0*) primers were designed using the National Center for Biotechnology Information website (Table 3).

### Synthesis of cDNA and gene expression analysis

Real-time PCR was performed with minor modifications of previously described methods described [10,11]. To synthesize cDNA, 1 μg of RNA was reverse transcribed in a 100 μL reaction volume with an iScript cDNA Synthesis Kit (Bio-Rad, Hercules, CA, USA) according to the manufacturer’s protocol. Quantitative real-time PCR (qRT-PCR) was performed on duplicate samples by using a CFX Connect Real-Time System (Bio-Rad, Seoul, Korea) with IQTM SYBR Green Supermix reagents (Bio-Rad, Seoul, Korea). The following PCR conditions were used: 95°C for 3 min and 40 cycles at 95°C for 10 s, 51°C to 65°C for 30 s and 72°C for 30 s. The mRNA levels of genes involved in myogenesis, adipogenesis, and preadipocyte determination and proliferation, including *MyoD*, *MYF5*, *MYF6*, *desmin*, *MyoG*, *C/EBPα*, *PPARγ*, *LPL*, *SCD*, *ERK1*, *ERK2*, *Wnt10B*, *β-catenin*, *Pref-1*, *Zfp423*, *Sox9*, and *KLF2*, were determined. *18S*, *GAPDH*, and *RPLP0* were used as housekeeping genes to normalize expression and minimize errors in the analysis.

### Statistical analysis

All statistical analyses were performed using SAS 9.4 (SAS Institute Inc., Cary, NC, USA). The experimental data were analyzed based on the mean serum VA, birth weight, and growth performance, and mRNA expression was assessed using independent (unpaired) samples t-tests for unequal variances. For the time effect in pregnant cattle, the mean serum VA values in each group were analyzed by (unpaired) sample t-tests and a generalized linear modeling procedure with Tukey’s test in the preliminary and main experiment, respectively. Significance was set at p≤0.05, and the tendency was declared at 0.05<p≤0.1.

## RESULTS AND DISCUSSION

### Effect of 78,000 IU vitamin A supplementation on serum vitamin A levels in pregnant cattle and the extended effect on newborn calves

**Figure 1:**
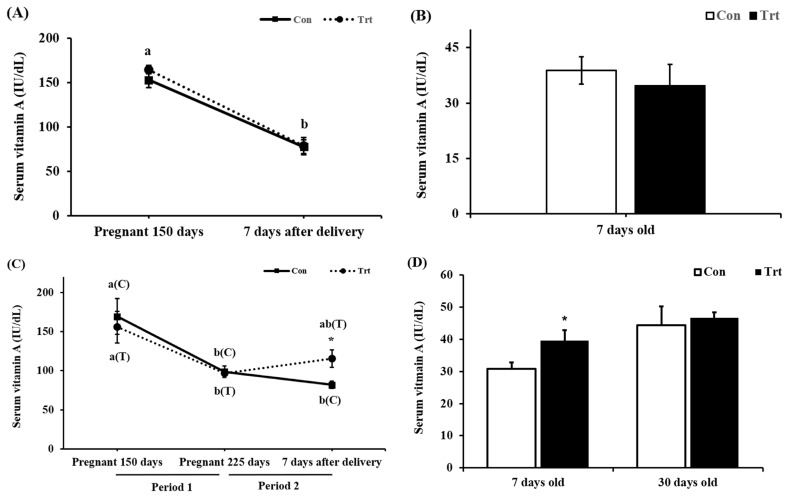
Changes in the concentration of serum vitamin A (VA) through VA supplementation in pregnant cattle and in calves. (A), (B) In the preliminary experiment, changes in serum VA (IU/dL) through the addition of VA (24,000 IU/d) for 75 days in pregnant cattle. (B) Serum VA levels in 7-day-old calves 3 control group (n = 18), ▪ treatment group (n = 8). (C), (D) In the main study, changes in serum VA (IU/dL) through the addition of VA (24,000 IU/d) from days 150 to 225 of gestation and 78,000 IU/d from day 225 until parturition in pregnant cattle. (C) In pregnant cattle, blood sampling was performed 3 times (pregnancy day 150, pregnancy day 225 and 7 days after delivery). (D) In calves, blood sampling was performed 2 times (age 7 days and age 31 days). 3 control group (n = 7), ▪ treatment group (n = 5). Mean±standard error, * p<0.05 indicates a significant difference between the groups. ^a,b^ Indicate that the means of each group are significantly different among the experimental period, (C) control group, and (T) treatment group.

In the preliminary experiment, no difference was observed in serum VA levels in pregnant cattle (p = 0.516; Figure 1A) 7 days after delivery or in newborn calves (p = 0.553; Figure 1B). In addition, we found a decreased serum VA levels in pregnant cattle (p<0.001; Figure 1A) after parturition both in the control and treatment groups. This phenomenon may indicate that pregnant cattle need higher levels of VA than those suggested by the Nutrient Requirements of Beef cattle (NRC [12]) and KFS [9] or may need 24,000 IU/d VA supplementation during late pregnancy, close to delivery. A previous study showed the same pattern, in which serum VA concentrations decreased in cattle during late pregnancy to support fetal growth [13,14].

When ingested VA is absorbed in mice, 66% to 75% goes to the liver through the blood vessels and can be stored in the form of retinyl ester, and 25% to 33% is transferred to peripheral tissues [15]. A fetus grows more rapidly after 150 days of gestation, and the fetus requires more nutrients, including VA, for growth [16]. Thus, the ratio of VA supplied to the fetus increase, and 25% to 33% of VA is transported to peripheral tissue. On 0 day, we found that mid-pregnant cows had an average serum VA concentration 152.7 IU/dL, which is considered within the a normal range in blood (84 to 200 IU/dL) [17]. However, the serum VA levels in our study were below the normal range at 7 days after delivery in both groups in the preliminary experiment, at 225 days of pregnancy in both groups in the main experiment, and at 7 days after delivery in the control group in the main experiment (p<0.05). Although the VA content in the basal diet was 21,606 IU/kg DM, it satisfied the NRC and KFS [9,12]. All mammals tend to maintain homeostasis, but few pregnant cows could remain within the normal VA range. A previous study showed a positive correlation (r^2^ = 0.77) between the VA stored in the liver and VA concentration in the blood [18]. Although the amount of VA stored in the liver was not confirmed in this experiment, decreased blood VA levels may indicate decreased VA levels stored in the liver. The control group was provided with 21,606 IU/kg VA in the basal diet, and this amount was based on the NRC and KFS guidelines for VA [9,12]. The serum VA concentration decreased in both the preliminary and main experiments. Thus, further studies are is needed to examine whether current requirements (NRC and KFS) are insufficient. However, the treatment group supplied with 24,000 IU/d VA showed no change in the serum VA level, perhaps because VA could not be stored in the liver and thus was directly supplied to and used by peripheral tissue, including the fetus. Therefore, we decided to increase the level of VA to 78,000 IU/d in pregnant cattle from day 225 of pregnancy until parturition (period 2) to clearly investigate the effects of supplemented VA. This level of VA was within the safe range of below 500,000 IU/d/head [19] to avoid toxicity. In the main experiment, the serum VA level in both groups dropped (p<0.01) during pregnancy whereas no significant difference (p = 0.839; Figure 1C) was observed between the two groups at day 225 of pregnancy. However, a significant increase in the serum VA level of the treatment group was observed 7 days after parturition (p = 0.021; Figure 1C). Vitamin A supplementation at 78,000 IU/d recovered blood VA levels to 120.3 IU/dL in the treatment group, which was higher than the value in the control group (83.7 IU/dL) (Figure 1C). Cattle were protected from the unexpected VA decrease during a critical time in the development of fetal organs [20]. Pregnant cattle have a greater need than nonpregnant cattle for different nutrients to complete normal body development. Furthermore, the serum VA concentration in the newborn calves showed a significant increase in the treatment group (p = 0.033, Figure 1D). The newborn calves receive VA through the placenta but not the colostrum; thus, we provided commercial colostrum. However, there was no difference in the serum VA level in 31-day-old calves (p = 0.719; Figure 1D). The level of VA supplementation in pregnant cattle may increase serum VA levels during birth but has no effect on subsequent serum VA levels in newborn calves.

### Effect of vitamin A supplementation in pregnant cattle on the growth performance of newborn calves

In the current study, we found that high VA supplementation in the preliminary experiment of 24,000 IU per day from day 225 of pregnancy led to an increasing trend in the birth weight of offspring (p = 0.088; Table 4). When the VA supplementation strategy increased to 24,000 IU/d from day 150 of pregnancy to day 225 (period 1) and to 78,000 IU/d from day 225 of pregnancy until parturition (period 2), we found an increased birth weight of the offspring of cattle that received with extra dietary VA supplementation in the main experiment (p = 0.022, Table 4). The addition of VA (78,000 IU/d) during late pregnancy had a preferable effect on postnatal birth weight; in the preliminary experiment, treated offspring were 3.2 kg heavier (p = 0.088) than untreated offspring, but, in the main experiment, treated offspring were 4.2 kg heavier (p = 0.022, Table 4) than untreated offspring. However, we did not obtain a balanced sex ratio in these two studies. Previous research showed that bull calves are approximately 1.9 to 2.3 kg heavier than heifer calves [21], but data in the current study showed no significant difference in weight between bull calves and heifer calves (p = 0.145) in the preliminary experiment (data not shown in the table). The results indicated that VA positively affected fetal growth during pregnancy. However, the BW of 31-day-old calves, feed intake, and gain-to-feed ratio were not different between the two groups (p>0.05). It was determined that the effects of VA supplementation were not sustained long enough to affect growth performance after birth; we did not proceed with VA supplementation after birth. Thus, further studies are needed to confirm the effect of VA supplementation on postnatal Korean native calves.

### Effect of high vitamin A supplementation in pregnant cattle on myogenesis in newborn calves

**Figure 2:**
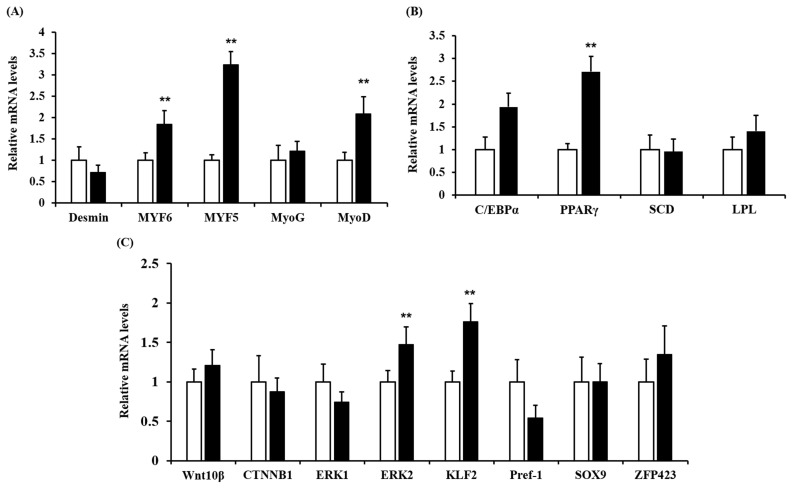
Gene expression study in offspring of vitamin A (VA)-treated pregnant cattle. Quantitative polymerase chain reaction (qPCR) validation of 18 genes in *longissimus dorsi* muscles of 31-day-old calves. The qPCR values are shown as the fold change in expression after normalization to the control genes 18S rRNA, glyceraldehyde-3-phosphate dehydrogenase, and ribosomal protein lateral stalk subunit P0. □, control group (n = 4), ▪, treatment group (n = 4). Data are presented as the means±standard error. *Desmin*, desmin; *MYF5*, myogenic factor 5; *MYF6*, myogenic factor 5; *MyoG*, myogenin; *MyoD*, myoblast determination protein; *SCD*, stearoyl-CoA desaturase; *C/EBPα*, CCAAT/enhancer-binding protein alpha; *LPL*, lipoprotein lipase; *PPARγ*, peroxisome proliferator-activated receptor gamma; *Wnt10B*, wnt family member 10B; *β-catenin*, beta-catenin; *ERK1/2*, extracellular signal-regulated kinase 1/2; *KLF2*, Krüppel-like factor 2; *Pref-1*, preadipocyte factor-1; *SOX9*, sox9; *ZFP423*, zinc finger protein 423. ** p<0.01; n = 4.

The relative mRNA expression of myogenic-related growth factors was greater in the offspring of the treatment group than in the offspring of the control group; included *MYF5* (p = 0.003), *MYF6* (p<0.001) and *MyoD* (p = 0.002; Figure 2A). In contrast, no differences were observed in the expression of *desmin* (p = 0.185) or *MyoG* (p = 0.382; Figure 2A) between the two groups. Uezumi et al [22] reported that myocytes and adipocytes are originally derived from the same pool of multipotent progenitor cells. Moreover, retinoic acid was reported to activate the *Wnt*/*β-catenin* pathway by stabilizing and enhancing the transcriptional activity of *β-catenin* [23]; in particular, *MYF5* expression was increased by 3.2-fold, and the expression of this gene is thought to be regulated by the *Wnt*/*β-catenin* pathway. The *Wnt*/*β-catenin* levels pathway may activate *MYF5* [24]. However, *Wnt10B* and *β-catenin* were not different between the control and treatment groups (p>0.05) (Figure 2C). It is possible that VA stimulated the expression of growth hormone genes such as thyroid (T3, T4) and glucocorticoid hormones to influence muscle growth [25]; not all of these hormones were measured in the current study. Hence, more research needs to be carried out to investigate the relationship between growth hormone genes and VA in muscle development.

In addition, a previous study showed that primary muscle fibers form in the bovine fetus within the first two months of conception and that the majority of muscle fibers form during the secondary myogenesis that occurs in the fetal stage between two and eight months of gestation in pregnant cattle [26]. Similarly, another previous study in bovine revealed that the number of muscle fibers continues to increase until the time around birth [27]. Therefore, in our study, the expression of *MYF5*, *MYF6*, and *MyoD* in the *longissimus dorsi* muscles of 31-day-old calves may positively affect muscle differentiation under the influence of maternal VA supplementation but will not affect the proliferation of myocytes, according to the aforementioned theory. These results suggest that VA supplementation contributed to the muscle differentiation via hypertrophy.

### Effects of high vitamin A supplementation in pregnant cattle on the development of preadipocytes in newborn calves

For adipocyte-related genes, *KLF2* (p<0.001) and *ERK2* (p = 0.004) levels were higher in the treatment group than in the control group (Figure 2). The *KLF2* and *ERK2* genes are critical in the embryogenesis stage and adipogenesis. *KLF2* has also been demonstrated to maintain the preadipocyte condition rather than promote adipogenic commitment [28]. In addition, 3T3-L1 cells overexpressing KLF2 showed reduced gene expression of *PPARγ* and *C/EBPα*, which are related to adipogenesis [28]. However, in the current study, both *KLF2* and *PPARγ* were expressed a higher level (p<0.05) in the VA treatment group than in the control group. *KLF2* is highly expressed in the preadipocyte period and is rapidly downregulated upon the induction of adipogenic differentiation [29]. Based on a previous study, we considered that *KLF2* gene expression indicates cells to maintain the preadipocyte state. *PPARγ* has been reported to play an essential role in adipogenesis in adipocytes [30] and a key role in the development of muscle by regulating of the insulin sensitivity and the transition of myosin heavy chain isoforms [31]. *Longissimus dorsi* muscle contains many types of cells, including muscle cells, preadipocytes, progenitor cells and immunocytes. Especially in the early growth stage in calves, *longissimus dorsi* muscle contains more muscle cells than other cell types. *PPARγ* is expressed in human skeletal muscle, although this expression level is two-thirds of that in adipocytes [32]. Amin et al [33] reported that the overexpression of *PPARγ* in murine muscle cells affects muscle fiber-type composition, lipid metabolism, and the synthesis and secretion of adiponectin (such as fatty acid oxidation), as well as improves muscle insulin sensitivity. Therefore, the upregulated *PPARγ* expression in the VA treatment group seemed to be related more to muscle development (corresponding to the increased gene expression of *MyoD*, *MYF5*, and *MYF6*, p<0.05) than to terminal adipogenic differentiation. Taken together, these data showing increased *KLF2* and *PPARγ* gene expression levels in the VA treatment group suggest that VA supplementation may help maintain the preadipocyte status and promote muscle development in the calf. Activation of the *ERK* pathway (*ERK1* and *ERK2*) promots adipogenic commitment in mouse embryonic stem cells upon retinoic acid supplementation [34], indicating that additional VA supplementation at 78,000 IU/d in pregnant cattle may increase the preadipocyte development of offspring. However, no differences were observed in the expression of *Wnt10B* (p = 0.054), *β-catenin* (p = 0.131), *SOX9* (p = 0.683), *Pref-1* (p = 0.275), *ERK1* (p = 0.097), or *Zfp423* (p = 0.201; Figure 2B). In a previous study, *Pref-1* and *Wnt10B* were used as potential markers of preadipocytes [35]. In addition, Berry et al [36] reported that high VA supplementation in 8-week-old male mice for 8 weeks and retinoic acid treatment in preadipocytes resulted in increased *Pref-1*, *Sox9*, and *KLF2* gene expression levels both *in vivo* and *in vitro*. Moreover, *Zfp423* was reported to be highly expressed in preadipocytes (within adipogenic epithelial cells) and mature adipocytes (brown adipocytes and white adipocytes), which is also related to preadipocyte proliferation and adipogenic differentiation [6]. Furthermore, retinoic acid reduces DNA methylation at CpG-rich promoters, such as the *Zfp423* promotor, that are primarily regulated by epigenetic modifications [6]. However, in the current study, we did not find any significant changes in the expression of *Pref-1*, *Sox9*, *ERK1*, or *Zfp423*.

The mRNA expression of a genes related to late adipogen esis, such as *LPL*, *C/EBPα*, and *SCD*, was not show different between the two groups (p>0.05, Figure 2B). C/EBPα, LPL, and SCD are used as marker genes for mature adipocytes and are considered as important factors for stimulating adipogenic differentiation and lipid accumulation in mature adipocytes [3]. We hypothesized that VA supplementation inhibited adipogenic differentiation, but *longissimus dorsi* muscle in calves contain few mature adipocytes.

## CONCLUSION

Vitamin A supplementation was administered to pregnant cattle from days 150 or 225 of pregnancy until parturition in our study. Our results suggested that a high level of VA supplementation (78,000 IU/d) during late pregnancy increases the birth weight of offspring; however, there was no significant increase in later growth performance. For gene expression in the 31-day-old calves, increased levels of *MYF5*, *MYF6*, *MyoD*, and *PPARγ* were observed in the muscles of the VA treatment group, while increased levels of *KLF2* and *ERK2* were observed in the VA-treated group, indicated potential development. Although this experiment showed that VA supplementation in the fetal stage can change gene expression in the *longissimus dorsi* muscles of calves, there is insufficient evidence of an increase in preadipocytes. Overall, to examine an increase in number of preadipocytes, future studies should investigate the histology of preadipocytes or mature adipocytes.

## Figures and Tables

**Table 1:** Composition and nutrient content of experimental diets for pregnant cattle (as-fed basis)*

Items	Dietary treatment1)		

	Basal	Supplementation2)	

		Period 1	Period 2
Ingredients (%)			
Pineapple core and rind	44.3	44.3	44.3
Rice straw	33.2	33.2	33.2
Base feed3)	11.1	11.1	11.1
Cottonseed	5.5	5.5	5.5
Alfalfa	2.8	2.8	2.8
Wheat flour	1.1	1.1	1.1
Bean flour	1.1	1.1	1.1
Azomite4)	0.6	0.6	0.6
Vitamin mixture5)	0.1	0.1	0.1
Calcium carbonate	0.2	0.2	0.2
Vitamin A supplementation (IU/50 g/d)6)	-	24,000	78,000
Analyzed composition (%)			
Water	62.3	62.3	62.3
Crude protein	4.0	4.0	4.0
Ether extraction	1.54	1.54	1.54
Crude fiber	9.41	9.41	9.41
Acid detergent fiber	13.6	13.6	13.6
Neutral detergent fiber	22.9	22.9	22.9
Crude ash	5.28	5.28	5.28
Ca	0.8	0.8	0.8
P	0.3	0.3	0.3
Vitamin A (IU/d)	21,606	45,606	99,606

DM, dry matter; VA, vitamin A; TMR, total mixed ration.

1) Thirteen kilograms of basal feed (as-fed basis) were supplied in the daily diet; the feed was 4.9 kg of DM. As a result, the composition level was similar to that required by the NRC [12]. The control and treatment groups consumed the same basal feed. VA supplementation was provided to pregnant cattle for 75 days. The amount of supplementation was very small (50 g) compared to that in the basal feedstuff (as-fed basis, 0.38%) and did not affect the energy value.

2) Supplementation: Period 1, days 150 to 225 of gestation, VA 24,000 IU/50 g; Period 2, day 225 of gestation to delivery, VA 78,000 IU/50 g.

3) Basal feedstuff: CP 18%, EE 4.7%, CF 20%, CA 12%, Ca 0.8%, P 1.6%.

4) Supplied per kilogram of complete diet: soluble potash (K2O) 120 mg, calcium (Ca) 108 mg, magnesium (Mg) 30 mg and sodium 6 mg.

5) Supplied per kilogram of complete diet: vitamin A 1,662 IU, vitamin D_3_ 332 IU, vitamin E 5.5 mg, vitamin K_3_ 2.2 mg, Mn 11 mg, Fe 11.1 mg, Cu 1.1 mg, Zn 11.1 mg, Se 0.2 mg and ethoxyquin 5.5 mg.

6) Composition of VA: 4,580 IU/g

* If a pregnant heifer weighs 400 kg and eats 7.85 kg of feed (DM basis), the VA requirement is 21,980 IU (2,800 IU×7.85 [kg, DM] = 21,980 IU/d). The total VA in the TMR (basal feedstuff) met NRC requirement [12].

**Table 2:** Composition and nutrient content of experimental diets for calves (as-fed basis)

Nutrient composition	Dietary treatment	

	Colostrum milk1)	Milk replacer2)
Moisture (%)	71.88	90.9
Crude protein (%)	14.92	2.0
Ether extraction (%)	6.7	1.6
Crude fiber (%)	-	0.5
Crude ash (%)	0.05	1.1
Calcium (%)	0.05	0.06
Phosphorus (%)	0.05	0.14
Retinyl acetate (IU/L)	-	2,275
Retinol (IU/L)	458,800	-
β-Carotene (IU/L)	76,500	-
Immunoglobulin G (g/L)	60	-

1) Colostrum milk (Headstart, Saskatoon Colostrum Company Ltd., Saskatoon, SK, Canada). This product is a dried colostrum, which is supplemented with IgG.

2) Milk replacer (Denkavit Co, Voorthuizen, Netherlands). The 1:10 dilution was recommended by the company.

**Table 3:** Primer sequences used in quantitative real-time polymerase chain reaction assays

Gene symbol	Gene name	Annealing temperature (°C)	Forward primer1) (5′→ 3′)
*Desmin*	Desmin	61.0	F: GGACCTGCTCAATGTCAAGA
			R: GGAAGTTGAGGGCAGAGAAG
*MYF5*	Myogenic factor 5	63.0	F: TGTCTTTAAGGTGGGGCTCA
			R: GAGTCCGCGAGATGCTATTC
*MYF6*	Myogenic factor 6	60.7	F: GAAGGAGGGACAAGCATTGA
			R: GAGGAAATGCTGTCCACGAT
*MYOD*	Myoblast determination protein	59.6	F: AGAGTTGCTTTGCCAGAG
			R: CTGCCTGCCGTATAAACA
*MYOG*	Myogenin	64.4	F: CAGGGCTCTTCTAAGCCAGG
			R: CAGAACTGCCCTCTTGCTCT
*C/EBPα*	CCAAT/enhancer-binding protein alpha	60.3	F: CCGTGGACAAGAACAGCAACGA
			R: GGCGGTCATTGTCACTGGTCAG
*LPL*	Lipoprotein lipase	60.0	F: TACCCTGCCTGAAGTTTCCAC
			R: CCCAGTTTCAGCCAGACTTTC
*SCD*	Stearoyl-CoA desaturase	60.0	F: TCCGACCTAAGAGCCGAGAA
			R: GCAGGATGAAGCACAACAACAG
*PPARγ*	Peroxisome proliferator-activated receptor gamma	61.0	F: TGGAGACCGCCCAGGTTTGC
			R: AGCTGGGAGGACTCGGGGTG
*ERK1*	Extracellular signal-regulated kinase 1	60.0	F: GGACCTGATGGAGACAGACCTGTA
			R: CGTTGGCGGAGTGGATATACTTCA
*ERK2*	Extracellular signal-regulated kinase 2	60.0	F: AACAAAGTCCGAGTCGCCAT
			R: CGATGGTCGGTGCTCGAATA
*KLF2*	Krüppel-like factor 2	62.5	F: ATTAAGCGTCGTCTTCCCCC
			R: ACCAGGTAGTCAAAATGCCCA
*Sox9*	Sox9	64.6	F: CTGAAGAAGGAGAGCGAGGAGGAC
			R: GCTTGACGTGCGGCTTGTTCT
*Pref-1*	Preadipocyte factor-1	58.8	F: CCTCTTGCTCCTGCTGGCTTTC
			R: AAGGTCACGCACTGGTCACAC
*β-Catenin*	Beta-catenin	63.8	F: AACACAGCAGCAGTTTGTGG
			R: CCTCTGATAACGATTCGGTTG
*Wnt10B*	Wnt family member 10 B	63.9	F: TTGATACTCACAACCGCAACTCCG
			R: TTGATACTCACAACCGCAACTCCG
*18S*	18S ribosomal RNA	51.0	F: ACCCATTCGAACGTCTGCCCTATT
			R: TCCTTGGATTGTGGTAGCCGTTTCT
*GAPDH*	Glyceraldehyde-3-phosphate dehydrogenase	60.0	F: GGCAAGGTCATCCCTGAG
			R: GCAGGTCAGATCCACAACAG
*RPLP0*	Ribosomal protein lateral stalk subunit P0	55.0	F: CAACCCTGAAGTGCTTGACAT
			R: AGGCAGATGGATCAGCCA

1) F, forward; R, reverse.

**Table 4:** Growth performance of newborn calves under vitamin A supplementation from gestational days 225 until parturition (the preliminary experiment) or from gestational days 150 until parturition (the main experiment)

Items	Group		SEM	p-value

	Control	Treatment		
Birth weight (kg)				
Preliminary experiment1)	25.9	29.1	1.07	0.088
Main experiment2)	21.8	26.0	1.56	0.022
Growth performance2)				
Body weight of 1-month-old (kg)	25.7	28.7	1.83	0.124
Feed intake (dry matter basis, kg/d)	0.23	0.24	0.02	0.568
Vitamin A intake (IU/d)	5,723	6,003	399	0.568
Average daily gain (kg/d)	0.33	0.41	0.05	0.324
Gain to feed ratio (kg/kg)	0.51	0.41	0.10	0.404

SEM, standard error of the mean.

1) Control: n = 18 (7 bulls and 11 heifers); treatment: n = 8 (4 bulls and 4 heifers).

2) Control: n = 7 (0 bulls and 7 heifers); treatment: n = 5 (3 bulls and 2 heifers).
